# Chronic anterior glenohumeral instability in soccer players: results for a series of 28 shoulders treated with the Latarjet procedure

**DOI:** 10.1007/s10195-012-0201-3

**Published:** 2012-07-01

**Authors:** Simone Cerciello, Thomas Bradley Edwards, Gilles Walch

**Affiliations:** 1 Centre Orthopedique Santy, 24, Avenue Paul Santy, 69008 Lyon, France; 2 Fondren Orthopedic Group, Texas Orthopedic Hospital, 7401 Main Street, Houston, TX 77030 USA

**Keywords:** Glenohumeral instability, Bone loss, Soccer, Latarjet procedure, Bankart repair

## Abstract

**Background:**

Glenohumeral instability is a common problem in young and active patients. Both open and arthroscopic procedures have proven to be effective options. In cases with large bone defects on the glenoid side or on the humeral head or in contact sports, arthroscopy leads to a high risk of recurrence. We report the results of the modified Latarjet procedure in a population of 26 soccer players affected by chronic anterior instability. To our knowledge there are no previous reports on the results of this procedure when used in a homogeneous group of sportsmen.

**Materials and methods:**

Twenty-six patients (28 shoulders) were retrospectively reviewed. We analyzed the roles of the players, the levels at which they played, and the average amount of hours that they trained before their injury and after surgery. Moreover, the type of bone loss detected on a preoperative imaging study and its relevance to the patient’s sporting comeback was recorded.

**Results:**

Eight-five months after surgery the mean Duplay score was 89.3; most of the players came back to the play at the same sporting level. Ninety-three percent of the patients were happy or very happy with their functional results. One patient underwent a redislocation.

**Conclusions:**

Our series is the first in the literature to refer to a homogeneous group of soccer players. According to our results, and other series, the Latarjet procedure seems to be the gold standard in the treatment of chronic anterior instability in patients with large bone defects and in sportsmen playing contact sports.

## Introduction

Chronic anterior glenohumeral instability is a controversial issue in orthopedic surgery. Bony transfers, capsular shifts, and labral repairs using both arthroscopic and open approaches have been described in the past. The treatment of athletes is even more complex, especially for those who play contact sports such as rugby, soccer, or basketball, in which players suffer higher-energy injuries.

Some articles have reported good results in the treatment of anterior glenohumeral instability using the Latarjet procedure in rugby players [1]. The efficacy of this procedure has been initially attributed to the bone block, which increases the anteroposterior diameter of the glenoid. However, Patte stressed the other two effects of this procedure, proposing the term “triple blocking”. The first action is due to the bone block, which increases the glenoid diameter. The second is attributed to the fibers of the inferior third of the subscapularis, which pulls posteriorly on the humeral head in abduction–external rotation. The third effect is due to the restoration of the anteroinferior capsular wall through the suture of the lateral capsular flap to the medial centimeter of the coracoacromial ligament, which remains attached to the coracoid process.

The procedure used in the present work was modified by the senior surgeon by performing a stable fixation with two malleolar screws and preserving the integrity of the fibers of the subscapularis tendon [2]. These two actions allow the surgeon to immediately begin rehabilitation with no limitation on the external rotation. This procedure has been demonstrated to be safe and reliable in athletes [1]. In fact, the results of these open procedures appear to be superior to those of arthroscopic procedures when compared in the high-risk athlete [3]. While the risk of recurrent instability is lower overall in soccer players than in rugby players, it is actually higher in goalkeepers.

The aim of this study was to evaluate the results of our application of the Latarjet procedure to the treatment of chronic anterior glenohumeral instability in soccer players, paying particular attention to the results for goalkeepers. Specific outcomes investigated were the delay in return to sporting activity, the postoperative level of activity, and the role (e.g., goalkeeper, defender, etc.) played after surgery.

## Materials and methods

We performed a retrospective study of a population of 46 soccer players (51 shoulders). Twenty-six patients (28 shoulders) were re-evaluated both clinically and radiographically. Mean follow up was 85 months (5–180). All patients were male.

The right shoulder was involved in 15 cases (53.7 %), and the dominant arm was affected in 13 patients (46.4 %). Patients suffered an average of 7.5 (1–50) dislocations before deciding to undergo the surgical procedure. Mean age at the time of first dislocation was 21 years (15–32). The first dislocation occurred while playing soccer in 23 cases (82 %); while diving in two cases (7.2 %); in a motor vehicle accident in one case (3.6 %); while skiing in one case (3.6 %); and while riding a bike in one case (3.6 %). The most frequent mechanism of injury was a fall on the shoulder or elbow while jumping during a game.

A supraspinatus lesion diagnosed with an arthro-CT scan was associated with the anterior instability in one patient. Two transient axillary nerve palsies were reported.

All but three patients played soccer at a semi-professional or professional level.

They performed an average of 10 h of training weekly. Seven players (eight shoulders) were goalkeepers; eleven were defenders, four (five shoulders) were midfielders, and four were forwards. Preoperative X-ray evaluation consisted of double obliquity anterior–posterior (AP) films in neutral internal and external rotations and a bilateral Bernageau film for the glenoid rim. A computed tomography (CT) scan was not performed routinely.

**Fig. 1:**
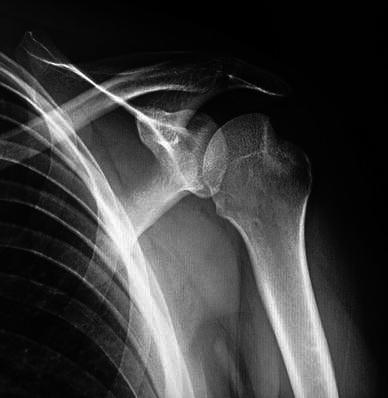
The presence of a large Hill–Sachs lesion is well detected in internal rotation and is an indication for the open Latarjet procedure

**Fig. 2:**
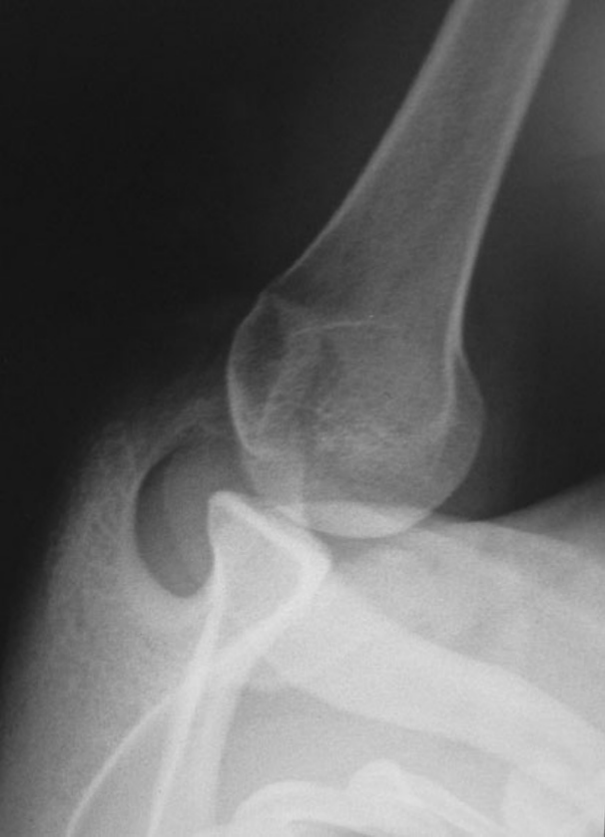
Bernageau view shows the exact anterior contour of the glenoid socket. Blunted angle sign refers to the loss of the sharp profile of the anteroinferior glenoid rim

**Fig. 3:**
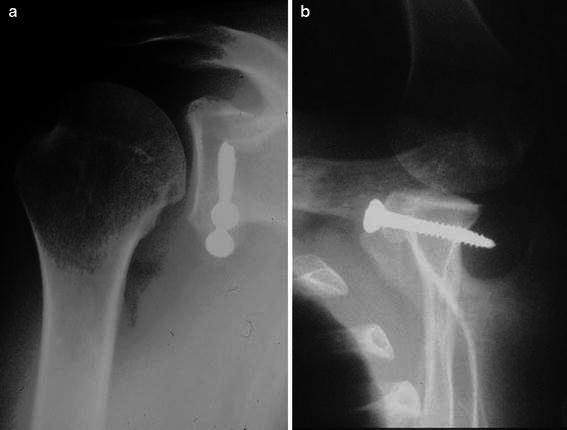
Postoperative X-ray control: AP view (**a**) and Bernageau view (**b**), showing that the graft has perfect positioning, with no overhang into the joint

AP film in internal rotation was particularly useful for detecting Hill–Sachs lesions of the posterior–superior aspect of the humeral head, while Bernageau’s film offered a precise view of the anteroinferior glenoid rim, allowing the detection of bone defects or fractures at this site. Bony Bankart lesions were thus classified according to their aspect into three types: fractures, cliff signs, and blunted angle signs (Figs.1,2,3).

Bony lesions were present in 22 cases (78.6 %), a glenoid fracture was present in eight cases (36.4 %), a Hill–Sachs lesion was present in five shoulders (22.7 %), a blunted sign in five cases (22.7 %), and a Hill–Sachs lesion associated with a glenoid fracture or a blunted angle sign in two cases (9.1 %) and two cases (9.1 %), respectively. Surgery was performed at an average of 23 months (3–72) after the first dislocation.

For the population of goalkeepers, the mean age at the time of first dislocation was 19 (23–16) years; the right side was involved in six cases and the dominant side was involved in six cases. Average number of dislocations was 2.2 (1–3). All patients were professional or semiprofessional. Mean training time was 14 h weekly. Two glenoid fractures and a Hill–Sachs lesion plus a glenoid fracture were found in three (37.5 %) patients, while no bony lesion was detected in five (62.5 %) patients. Surgery was performed at an average of nine months (1–25) after the first dislocation (Table 1).

**Table 1:** Demographic data

	General population	Goalkeepers
Age	21 ± 4.7	19 ± 2.8
Dominant arm	46.4 %	75 %
Hours of training (weekly)	10 ± 6.8	14 ± 9.4
Number of dislocations	7.5 ± 11.3	2.2 ± 0.7
Bony lesions	78.6 %	37.5 %

The surgical steps for this procedure have been described previously by the senior author [2]. However, it is important to stress some concepts. First of all, this open procedure has a very low impact on soft tissues since we avoid releasing the subscapularis and perform a horizontal split of its fibers at the junction of the superior one-third with the inferior two-thirds. This avoids fatty degeneration of the subscapularis muscle, as was demonstrated by Maynou [4].

The coracoid is prepared with a 3.2 mm drill to reduce the risk of fracture, and fixed with two 4.5 mm malleolar screws to achieve optimal compression of the two bony surfaces. This is crucial to achieve optimal initial stability of the bone block, which is mandatory in order to avoid the risk of pseudarthrosis and thus realize optimal outcomes [2]. For those reasons, it is now performed as an outpatient procedure.

Postoperative care is relatively easy, with patients having their arm in a sling for 15 days. A rehabilitation program is started on the first postoperative day with passive exercises twice daily aimed at recovering the complete range of motion. At day 15, patients start swimming pool and progressive strengthening exercises. At three months postoperatively, all sports activities are allowed after thorough clinical and X-ray exams. Final follow-up was performed at an average of 85 months (5–180) after surgery with complete imaging analysis and clinical evaluation. Patients filled out a questionnaire consisting of two parts. The first determined the level of return to sport reached after surgery (delay, hours of training, level, role), the presence of re-dislocation, and the discomfort or pain during sports activities. The second was the Duplay score for anterior glenohumeral instabilities [2]. Overall objective scores were on a scale of 100; results from 100 to 90 were considered excellent, from 89 to 75 good, from 74 to 51 fair, below 50 poor. Patients’ subjective results were evaluated with the question: “About surgery, do you feel very happy, happy, disappointed or unhappy?”

This retrospective study was carried out according to the principles of the Declaration of Helsinki, and was approved by the local ethical committee. Moreover, all of the patients gave their informed consent before being enrolled for the evaluation.

## Results

Concerning the overall population, all but one of the patients came back to soccer with an average delay after surgery of eight months (2–24). Eighteen players (20 shoulders, 71.4 %) returned to play soccer at the same level as they did before surgery. Seven (25 %) players played soccer at a lower level; in two cases this was due to age- or job-related problems. One patient (3.6 %) changed sport. Four patients continued to feel discomfort when throwing the ball with the involved arm, while two patients were worried about having further traumas. One re-dislocation was noted in a goalkeeper 74 months after surgery. After surgery, the average training time was 8 h weekly. None of the players changed their roles. The average global Duplay score was 89.3: sport comeback score was 21.9, stability was 21.4, pain was 21, ROM was 25. Subjective results were good: 24 patients (86 %) were very happy, two patients (7 %) were happy, one (3.5 %) was disappointed, one (3.5 %) was unhappy. This last patient was the one who suffered a re-dislocation 74 months after surgery after a high-energy trauma in which he suffered axillary nerve palsy. No signs of arthritis were detected at the last X-ray follow-up.

The average delay among the goalkeepers before coming back after surgery was five months (3–8). All patients came back to sport at the same level as preoperatively, and none changed his role. One patient referred to discomfort while throwing the ball with the affected arm. As previously mentioned, one patient in this group underwent a re-dislocation 74 months after surgery.

Average training time after surgery was 14 h. At the last follow-up, the average global Duplay score was 91.2: sport comeback score was 25, stability was 18.7, pain was 22.5, ROM was 25. Subjective results were as follows: five patients (71.4 %) were very happy, one (14.3 %) was happy, and one (14.3 %) was unhappy (Table 2).

**Table 2:** Postoperative results

	General population	Goalkeepers
Time taken to return to sporting activity	8 months	5 months
Played at the same level	71.4 %	100 %
Played at a lower level	25 %	/
Quit	3.6 %	/
Hours of training (weekly)	8 ± 7.0	14 ± 9.4
Apprehension	20 %	12.5 %
Re-dislocation	/	12.5 %
Duplay score	89.3 ± 17.0	91.2 ± 21.0
Subjective results		
Very happy	86 %	91.2 %
Happy	7 %	14.3 %
Unhappy	3.5 %	14.3 %
Disappointed	3.5 %	/

## Discussion

Anterior glenohumeral dislocation and subsequent chronic anterior instability are common situations in orthopedic practice. In the past few decades, several studies have reported the results of both arthroscopic and open techniques. A recent meta-analysis compared the results of open and arthroscopic Bankart repair, and found no difference in terms of recurrence of instability (6.7 % and 6 %, respectively) and rate of reoperation (6.6 and 4.7 %) [5]. Concerning data after 2002, there was a recurrence rate of 2.9 % and a reoperation rate of 2.2 % in the arthroscopic group, compared with 9.2 % and 9.2 %, respectively, in the open group [5]. The arthroscopic Bankart repair has recently become a common option for the majority of surgeons due to improvements in suture anchors [6], and cosmesis as well as the possibility of identifying and treating additional intra-articular lesions such as HAGL, SLAP, or posterior Bankart lesions [7, 8].

The major disadvantage of arthroscopic Bankart repair is still the rate of recurrence. Recent studies have shown concerning data. Flinkkilä reports a recurrence rate of instability of 19 % (9 % from redislocation and 10 % from subluxation) and a revision surgery rate of 10 % [9], while Voos noted a recurrence rate of 18 % (10 % from redislocation and 8 % from subluxation) and a revision surgery rate of 6 % [10].

The risk of recurrence seems to be higher under specific conditions [1, 9, 11]. The age of the patient at the time of surgery is a risk factor [9]. The presence of bony lesions has a dramatic impact on surgical outcome, and its role in the development of recurrence has been stressed in several studies [11, 12]. Seung-Ho Kim found a statistically significant correlation between recurrent instability following arthroscopic capsular shifts and the presence of glenoid bony lesions involving more than 30 % of the glenoid surface [13]. Similar concerns are reported even in the case of open Bankart repair [14]. Clinical evidence stresses the need for bony transfers in the case of severe glenoid bone loss [15].

Athletes participating in contact sports are at a higher risk of recurrence. Several studies of rugby players have shown better results and a lower re-dislocation rate with open surgery than with arthroscopic procedures [1]. Recently, Balg proposed an “instability severity index score” that analyzes several risk factors in an attempt to identify patients at risk of re-dislocation after arthroscopic Bankart repair [16]. When the score was higher than six points, the recurrence rate reached 70 % and the Latarjet procedure was indicated. Although an arthroscopic technique for the Latarjet procedure was recently described by Lafosse, who reports excellent results [17], we prefer the open technique, which in our opinion is easier and more reliable.

Our population consisted of 26 soccer players (28 shoulders), which is a sport associated with a relatively high risk for glenohumeral instability. Falling directly on the shoulder or elbow are the most common causes of acute dislocation or subluxation. Glenohumeral instability is an important injury for defenders, midfielders, or forwards, but it usually does not preclude them from a return to a high level of competition. However, this injury can prove more problematic for goalkeepers. Recurrent instability is considered to be more frequent; persistent pain or residual apprehension can influence the player’s desire to dive and throw the ball (abduction and external rotation), limiting his performance. Surgical technique must address more demanding situations: early comeback after surgery, more stress on the repair, and less time to rest after matches and training. We strongly believe in the efficacy of the Latarjet procedure in the general population and even more so in athletes. The principles of this procedure have been explained by Patte with the “triple blocking” concept. The bony block is extremely important. As previously mentioned, the decrease of 25–30 % in the glenoid anterior–posterior diameter is the major cause of recurrence [11–14]. Burkhart reported the results of his modified Latarjet procedure in patients with severe bone loss, and noted that there were no recurrences and that only 2.2 % were apprehensive [18].

Several studies have reported the results of the Latarjet procedure, including good results and low recurrence rates [19–23]. Collin reviewed 69 patients at an average FU of 50 months, noting a satisfaction rate of 85 %, four re-dislocations, and two subluxations [19]. Hovelius, in a prospective study of 118 shoulders with 15 years of follow-up, found a satisfaction rate of 98 %, subluxation in 11 patients, and recurrence of instability in three patients [20]. Cassagnaud, in his series of 106 Latarjet procedures with 7.5 years of follow-up, reported that excellent results were obtained in 76.4 %, there was one re-dislocation, and there was a residual apprehension rate of 13.4 % [21]. Allain noted good results and no re-dislocations in a no-athlete population at an average follow-up of 14.3 years [22]. Doursounian reported a satisfaction rate of 92 % and one re-dislocation with his modified instrumentation [23]. Despite these excellent clinical and functional results, there are still some concerns about the adverse effects of the Latarjet procedure. The first is the functional and anatomic modifications of muscular structures related to the coracoid transfer. A recent study demonstrated that the Latarjet procedure does not modify the size and morphology of the biceps muscle [24]. The other historic concern is the onset of glenohumeral arthrosis years later [25]. It was reported at a SOFCOT symposium in 1999 that both the Latarjet procedure and Bankart repair showed the same evolution of arthrosis [26]. Allain, at a follow-up of 14.3 years, found correlations between arthrosis and associated cuff lesions, lateral overhang of the coracoid, and intra-articular screws [22]. Matsoukis found correlations between arthrosis and age at first dislocation as well as the presence of bony lesions on both the glenoid and humeral sides, while no correlation was found with the type of surgical technique [27]. It should be noted that arthrosis usually evolves rather slowly (an average of 28 years), and appears to continue whether the shoulder is stabilized or not.

According to these data, we can conclude that the Latarjet procedure is an established option in the treatment of chronic anterior instability. Our series is the only one in the literature concerning a homogeneous group of soccer players. This operation is particularly indicated in such patients, since it allows for a faster return to sport after surgery, and most patients regain their pre-injury level of performance. Objective and subjective results were very good. Four patients referred to discomfort when throwing the ball (overhead activity), and one patient (a goalkeeper) underwent a re-dislocation due to a high-energy event. No signs of arthritis were detected at the last X-ray follow-up. These results are still very satisfactory for this high-risk subpopulation of goalkeepers. All patients in this group showed a rapid return (five months) to the same sporting performance level and role after surgery. Our re-dislocation rate in this high-risk population was 3.5 %, while this risk among the goalkeepers increased to 12.5 %.

In our opinion, the Latarjet procedure is the gold standard in the treatment of chronic anterior glenohumeral instability in athletes.

## References

[1] Sonnery-Cottet B, Dagher E, Jouve F, Neyton L, Nové-Josserand L, Walch G (2004) Instabilité anterieure de l’épaule chez le rugbyman, resultants d’une serie de 85 épaules operées par la technique de Latarjet avec un recul moyen de 7 ans. In: Pathologies du rugbyman. Sauramps Médical, Montpellier, pp 327–336

[2] Walch G (1991). La luxation récidivante antérieure de l’épaule. Rev Chir Orthop.

[3] Solhpour S, McMahon PJ (2003). Operative treatment of labral lesions: arthroscopic versus open treatment. Curr Opin Orthop.

[4] Manyou C, Cassagnaud X, Elise S, Mestdagh H (2002). Long-term study of the subscapularis after surgical treatment of recurrent shoulder instability using a screwed coracoid bone block. J Bone Joint Surg Br.

[5] Petrera M, Patella V, Patella S, Theodoropoulos J (2010). A meta-analysis of open versus arthroscopic Bankart repair using suture anchors. Knee Surg Sports Traumatol Arthrosc.

[6] Kocaoglu B, Guven O, Nalbantoglu U, Aydin N, Haklar U (2009). No difference between knotless sutures and suture anchors in arthroscopic repair of Bankart lesions in collision athletes. Knee Surg Sports Traumatol Arthrosc.

[7] Lino W, Belangero WD (2006). Labrum repair combined with arthroscopic reduction of capsular volume in shoulder instability. Int Orthop.

[8] Arrigoni P, Huberty D, Brady PC, Weber IC, Burkhart SS (2008). The value of arthroscopy before an open modified Latarjet reconstruction. Arthroscopy.

[9] Flinkkilä T, Hyvönen P, Ohtonen P, Leppilahti J (2010). Arthroscopic Bankart repair: results and risk factors of recurrence of instability. Knee Surg Sports Traumatol Arthrosc.

[10] Voos JE, Livermore RW, Feeley BT, Altchek DW, Williams RJ, Warren RF, Cordasco FA, Allen AA (2010). Prospective evaluation of arthroscopic Bankart repairs for anterior instability. Am J Sports Med.

[11] Burkhart SS, De Beer JF (2000) Traumatic glenohumeral bone defects and their relationship to failure of arthroscopic Bankart repairs: significance of the inverted-pear glenoid and the humeral engaging Hill–Sachs lesion. Arthroscopy 16:677–694 10.1053/jars.2000.17715 11027751

[12] Itoi E, Lee SB, Berglund LJ, Berge LL, An KN (2000). The effect of a glenoid defect on anteroinferior stability of the shoulder after Bankart repair: a cadaveric study. J Bone Joint Surg Am.

[13] Seung-ho K, Kwon-Ick HA, Yang-Bum C, Byung-Dam R, Irvin O (2003). Arthroscopic anterior stabilization of the shoulder: two to six years follow up. J Bone Joint Surg Am.

[14] Rhee YG, Lim CT (2007) Glenoid defect associated with anterior shoulder instability: results of open Bankart repair. Int Orthop 31(5):629–634 10.1007/s00264-006-0234-4 PMC2266647 17006660

[15] Churchill RS, Moskal MJ, Lippett SB, Matsen FAIII (2001). Extracapsular anatomically contoured anterior glenoid bone grafting for complex glenohumeral instability. Tech Shoulder Elbow Surg.

[16] Balg F, Boileau P (2007). The instability severity index score. A simple pre-operative score to select patients for arthroscopic or open shoulder stabilisation. J Bone Joint Surg Br.

[17] Lafosse L, Boyle S (2010) Arthroscopic Latarjet procedure. J Shoulder Elbow Surg 19(2 suppl):2–12 10.1016/j.jse.2009.12.010 20188263

[18] Burkhart SS, De Beer JF, Barth JR, Cresswell T, Roberts C, Richards DP (2007). Results of modified Latarjet reconstruction in patients with anteroinferior instability and significant bone loss. Arthroscopy.

[19] Collin P, Rochcongar P, Thomazeau H (2007). Treatment of chronic anterior shoulder instability using a coracoid bone block (Latarjet procedure): 74 cases. Rev Chir Orthop Reparatrice Appar Mot.

[20] Hovelius L, Sandström B, Sundgren K, Saebö M (2004) One hundred eighteen Bristow–Latarjet repairs for recurrent anterior dislocation of the shoulder prospectively followed for fifteen years: study I—clinical results. J Shoulder Elbow Surg 13(5):509–516 10.1016/j.jse.2004.02.013 15383806

[21] Cassagnaud X, Maynou C, Mestdagh H (2003) Clinical and computed tomography results of 106 Latarjet–Patte procedures at mean 7.5 year follow-up. Rev Chir Orthop Reparatrice Appar Mot 89(8):683–692 14726834

[22] Allain J, Goutallier D, Glorion C (1998). Long-term results of the Latarjet procedure for the treatment of anterior instability of the shoulder. J Bone Joint Surg Am.

[23] Doursounian L, Debet-Mejean A, Chetboun A, Nourissat G (2009) Bristow–Latarjet procedure with specific instrumentation: study of 34 cases. Int Orthop 33(4):1031–1036 10.1007/s00264-008-0606-z PMC2898983 18633611

[24] Castoldi F, Rossi R, Lollino N, Renzulli F, Berrino E, Rossi P (2008) Coracoid transfer in Bristow–Latarjet procedure: does it modify the biceps muscle? Knee Surg Sports Traumatol Arthrosc 16(1):81–85 10.1007/s00167-007-0436-3 17989955

[25] Young DC, Rockwood CA (1991). Complications of failed Bristow procedure and their management. J Bone Joint Surg Am.

[26] Coudane H, Walch G (2000). L’instabilité antérieure cronique del’épaule chez l’adulte. Rev Chir Orthop.

[27] Matsoukis J, Tabib W, Guiffault P, Mandelbaum A, Walch G, Nemoz C, Edwards TB (2003). Shoulder arthroplasty in patients with prior anterior shoulder dislocation. Results of a multicenter study. J Bone Joint Surg Am.

